# Oxidants and Antioxidants in Metabolic Syndrome and Cancer

**DOI:** 10.1155/2014/178962

**Published:** 2014-06-24

**Authors:** Si Jin, Yongzhong Wu, Shiwei Deng, Jinxiang Zhang, Xiao Qian Chen

**Affiliations:** ^1^Department of Pharmacology, Tongji Medical College, Huazhong University of Science and Technology, Wuhan 430030, China; ^2^Laboratory of Molecular Pharmacology, Oxidative Signaling and Molecular Therapeutics Section, Center for Cancer Research, National Cancer Institute, Bethesda, MD 20892, USA; ^3^Department of Pharmacology, University Medical Center of the Johannes Gutenberg University Mainz, Obere Zahlbacher Straße 67, 55131 Mainz, Germany; ^4^Department of Surgery, Union Hospital, Tongji Medical College, Huazhong University of Science and Technology, Wuhan 430022, China; ^5^Department of Pathophysiology, Tongji Medical College, Huazhong University of Science and Technology, Wuhan 430030, China

Metabolic syndrome, which includes hypertension, hyperlipidemia, obesity, insulin resistance or glucose intolerance, and fatty liver diseases, significantly contributes to the increased risk of cardio- or cerebral-vascular diseases and diabetes [1]. Cancer represents a group of malignantly proliferative diseases. Recently, metabolic reprogramming changes were extremely highlighted in cancer cells, putting forward to a trend that cancer should also be treated as a type of metabolic disease [2]. As a matter of fact, statins, the most prescribed low density lipoprotein (LDL) lowering agents, and metformin, the most prescribed antidiabetic drug, have both been reported to associate with decreased cancer risk [3, 4].

Meanwhile, in the past decade, a growing body of evidence has implicated that increased oxidative stress is a common and key feature of metabolic diseases [5]. Whereas a recent hypothesis paper by Watson, the famous Nobel Prize winner, suggests that diabetes, dementias, cardiovascular disease, and some cancers may be linked to a failure to generate sufficient reactive oxygen species (ROS) [6], which maybe reflects the situations in end stages of these complex diseases. ROS play very important roles in carcinogenesis and the maintenance of cancer cells. The ROS levels are reported to be very high in cancer cells, which drive the high rate of proliferation in these malignant cells [7]. Whether prooxidants or antioxidants could be used to treat cancer or not remains elusive.

In this controversial context, we organized this special issue.

Although insulin is critically involved in metabolism regulation and closely associates with both diabetes and cancer, the precise molecular mechanism is not fully elucidated. In this special issue, Q. Li et al. conducted an interesting study, which demonstrates that insulin increases pyruvate kinase M2 (PKM2) expression through ROS for the regulation of glucose consumption and lactate production “*Insulin regulates glucose consumption and lactate production through reactive oxygen species and pyruvate kinase M2*”. J. Zhao et al. demonstrated that pubertal obesity was associated with increased oxidative stress in testis tissue and high levels of leptin, which maybe the cause of male hypogonadism in obesity “*Leptin level and oxidative stress contribute to obesity-induced low testosterone in murine testicular tissue.*” Oxidized low density lipoprotein (oxLDL) is the major lipid found in atherosclerotic lesion and elevated plasma oxLDL is recognized to be a risk factor of atherosclerosis. W. Li et al. in this special issue emphasized the critical role of oxLDL transcytosis across endothelial cells in the initiation of atherosclerosis, providing novel insight into the pathogenesis of this complex metabolism disease “*Endogenous ceramide contributes to the transcytosis of oxLDL across endothelial cells and promotes its subendothelial retention in vascular wall.*” For the therapy of metabolic syndrome-related disorders, J. Wang et al. reported in this special issue that* amagnolia *extract, named BL153, could prevent obesity-induced liver damage via inhibition of lipid accumulation, inflammation, oxidative stress “*BL153 partially prevents high fat diet induced liver damage probably via inhibition of lipid accumulation, inflammation, and oxidative stress.*”* Centella asiatica *is a traditional Chinese medicine which has been reported to have antioxidant effect in vitro. In this special issue, Y. Zhao et al. further reported that* Centella asiatica* decreased cholesterol and triglyceride in mice model “*Effect of Centella asiatica on oxidative stress and lipid metabolism in hyperlipidemic animal models.”* Salidroside (SAL) is an active component of* Rhodiola Rosea *with documented antioxidative properties. S. Xing et al. in this issue further demonstrated that SAL could protect endothelium against H_2_O_2_-induced injury via promoting mitochondrial biogenesis and function, thus preventing the over-activation of oxidative stress-related downstream signaling pathways “*Salidroside stimulates mitochondrial biogenesis and protects against H*_*2*_ *O*_*2*_ *-Induced endothelial dysfunction.*”

In cancer cells, the ROS is maintained at higher levels than normal cells and mainly exerts its proliferative actions. When ROS levels are further increased by prooxidants so as to exceed a border line level, the proapoptotic effects of ROS may exceed its proliferative effects and cytotoxic effects display in cancer cells, whereas the ROS levels in normal cells remain below the border line level which is nontoxic to normal cells. In consistent with this view, several studies in this special issue reported chemicals could kill cancer cells by inducing ROS accumulation as cancer killing agents. Piperlongumine (PL) is a natural alkaloid from* Piper longum* L., possessing highly selective and effective anticancer property. L.-H. Gong et al. reported in this special issue that PL notably induced cell apoptosis, G2/M phase arrest and intracellular ROS accumulation in a dose- and time-dependent manner. Pretreatment with antioxidant N–acety-L-cysteine could reverse the PL-induced ROS accumulation and cellular apoptosis. In addition, low-dose of PL/cisplatin or paclitaxel combination therapies had a synergistic anti-growth effect on human ovarian cancer cells “*Piperlongumine induces apoptosis and synergizes with cisplatin or paclitaxel in human ovarian cancer cells.*” Q. R. Liu et al. also reported that Piperlongumine effectively inhibited the migration of human glioma cells but not normal astrocytes in the scratch-wound culture model. PL increased ROS production, reduced glutathione, activated p38 and JNK pathway, increased I*κ*B and suppressed NF-*κ*B in LN229 cells after scratching “*Piperlongumine inhibits migration of glioblastoma cells via activation of ROS-dependent p38 and JNK signaling pathways*”. Similarly, H. Li et al. also reported in this issue that Lithium chloride, the established glycogen synthase kinase-3*β* (GSK-3*β*) inhibitor, induced the apoptosis of colorectal cancer cells by induction of ROS signaling “*Lithium chloride suppresses colorectal cancer cell survival and proliferation through ROS/GSK-3*β*/NF-*κ*B signaling pathway.*”

In contrast to above observations which demonstrate that induction of ROS may be used to treat cancers, Y-Q. Xue et al., in this special issue reviews the preventive or anticancer properties of resveratrol oligomers. Resveratrol is a naturally derived phytoalexin stilbene isolated from grapes and other plants with documented antioxidant activity “*Resveratrol oligomers for the prevention and treatment of cancers.*” In line with this review, Y. Li et al. reported that NADPH oxidase 4 (Nox4)-derived reactive oxygen species might play crucial roles in the invasion, angiogenesis and radioresistance in glioblastoma. Inhibition of Nox4 by lentivirus-mediated small hairpin interfering RNA (shRNA) could be a strategy to overcome radioresistance and in turn improve the therapeutic efficacy “*Lentivirus-mediated Nox4 shRNA invasion and angiogenesis and enhances radiosensitivity in human glioblastoma.*” These studies suggest that in addition to prooxidant therapy, antioxidants may also be the right option to treat cancers as cancer inhibiting agents.

In addition to above mentioned pathological or pharmacological studies, Elizabeth Moreno-Arriola et al. present an interesting review which suggests that* Caenorhabditis elegans,* which may be one of the most famous model organisms, is also a suitable disease model reflecting the oxidative status in metabolic disorders, providing new approaches for studying metabolic diseases “*Caenorhabditis elegans: a useful model for studying metabolic disorders in which oxidative stress is a contributing factor.*”

From these collected studies in this special issue, we appear to come to the consensus that upregulated oxidative stress indeed promotes the development of both metabolic syndrome and cancer. Antioxidants are suitable choices for the prevention or treatment of metabolic syndrome-based cardiovascular diseases or diabetic mellitus, which are also classified as chronic nonresolving inflammatory diseases [8]. However, for the treatment of cancers, both prooxidants and antioxidants might work via different mechanisms, provided that the drug selectivity on targeted tissue is guaranteed. We hope that the studies and discussions gathered in this issue reflect the current trend in this area and could, to some extent, stimulate more in-depth studies in this somehow controversial field.
